# Evaluation of SNP Data from the *Malus* Infinium Array Identifies Challenges for Genetic Analysis of Complex Genomes of Polyploid Origin

**DOI:** 10.1371/journal.pone.0067407

**Published:** 2013-06-27

**Authors:** Michela Troggio, Nada Šurbanovski, Luca Bianco, Marco Moretto, Lara Giongo, Elisa Banchi, Roberto Viola, Felicdad Fernández Fernández, Fabrizio Costa, Riccardo Velasco, Alessandro Cestaro, Daniel James Sargent

**Affiliations:** 1 Centre for Research and Innovation, Fondazione Edmund Mach, San Michele all'Adige, Italy; 2 East Malling Research, East Malling, Kent, United Kingdom; Auburn University, United States of America

## Abstract

High throughput arrays for the simultaneous genotyping of thousands of single-nucleotide polymorphisms (SNPs) have made the rapid genetic characterisation of plant genomes and the development of saturated linkage maps a realistic prospect for many plant species of agronomic importance. However, the correct calling of SNP genotypes in divergent polyploid genomes using array technology can be problematic due to paralogy, and to divergence in probe sequences causing changes in probe binding efficiencies. An Illumina Infinium II whole-genome genotyping array was recently developed for the cultivated apple and used to develop a molecular linkage map for an apple rootstock progeny (M432), but a large proportion of segregating SNPs were not mapped in the progeny, due to unexpected genotype clustering patterns. To investigate the causes of this unexpected clustering we performed BLAST analysis of all probe sequences against the ‘Golden Delicious’ genome sequence and discovered evidence for paralogous annealing sites and probe sequence divergence for a high proportion of probes contained on the array. Following visual re-evaluation of the genotyping data generated for 8,788 SNPs for the M432 progeny using the array, we manually re-scored genotypes at 818 loci and mapped a further 797 markers to the M432 linkage map. The newly mapped markers included the majority of those that could not be mapped previously, as well as loci that were previously scored as monomorphic, but which segregated due to divergence leading to heterozygosity in probe annealing sites. An evaluation of the 8,788 probes in a diverse collection of *Malus* germplasm showed that more than half the probes returned genotype clustering patterns that were difficult or impossible to interpret reliably, highlighting implications for the use of the array in genome-wide association studies.

## Introduction

As genome and transcriptome sequence data become more accessible due to the availability of affordable second-generation sequencing technologies, the characterisation of single nucleotide polymorphisms (SNPs) has become routine. Thus, large genomic SNP data sets have been reported for many diverse plant species including grapevine (*Vitis vinifera* L. [1]), red raspberry (*Rubus ideaus* L. [2]), apple (*Malus pumila* L. Borkh. [3]), oilseed rape (*Brassica napus* L. [4]), *Citrus sp*p. [5], and globe artichoke (*Cynara cardunculus* L. [6]).

SNPs are rapidly becoming the molecular marker of choice for genetic analyses at the species level due to their unbiased abundance throughout eukaryotic genomes, their generally binary, co-dominant nature, and their relative conservation and thus transferability. As such, they have been applied to a diverse range of genetic studies in plants including pedigree analyses, assessment of genetic diversity [7], estimations of linkage disequilibrium decay [8], genetic linkage map construction [2], [3], [9], [10], and association mapping [11].

Recent advances in high throughput genotyping technologies have meant that it is now possible to develop assays to screen thousands, to hundreds of thousands of polymorphic SNP loci in a given genotype or population. Whole genome genotyping (WGG) assays permit the economical and reliable genotyping in a semi-automated system, that permits SNPs to be multiplexed to include many tens or hundreds of thousands of markers per assay [12]. Array-based platforms available include the GoldenGate and Infinium platforms from Illumina, and the Axiom array platform by Affymetrix. Such assays require a large initial investment in development costs, but once an array has been developed, the genotyping cost per SNP per individual is relatively low and data can be generated for large numbers of samples reliably and rapidly.

The Illumina Infinium II technology has been extensively applied to plant species and arrays have been developed for grapevine [13], maize (*Zea mays* L.) [14], peach (*Prunus persica* (L.) Batsch [15], potato (*Solanum tuberosum* L. [16]), sunflower (*Helianthus annus* L. [17]) and tomato (*Solanum lycopersicum* L. [18]). Recently an Infinium II WGG array containing 8,788 SNPs was developed for apple (*Malus pumila* Mill.) and pear (*Pyrus communis* L.) using re-sequencing data from 27 *Malus* genotypes [19]. The array, containing 7,867 *Malus* and 921 *Pyrus* SNPs was developed as part of the International RosBREED SNP Consortium, and hence is denoted IRSC. The array was subsequently used to construct a comprehensive genetic linkage map for an apple rootstock mapping progeny M432 (‘M.27′×‘M.116′) by Antanaviciute et al [20]. In that study, a total of 2,856 heterozygous segregating markers were identified, 2,272 of which were incorporated into a consensus genetic linkage map for the progeny [20]. The authors reported that the remaining 584 segregating loci were not located to LGs on the M432 map due to poor separation of clusters called automatically by GenomeStudio (Illumina).

*Malus pumila* Mill. is a diploid species (2*n* = 2*x* = 34), however, the extant genome of the species is known to have been derived from an ancient whole genome duplication (WGD) event [3]. The recent sequencing of the genome of the cultivar ‘Golden Delicious’ [3] demonstrated that the majority of the chromosomal homeologues are still detectable in *Malus* genome sequence, and that an unprecedented number of paralogous gene loci exist within the apple genome. The Infinium array technology generates a signal for SNP calling from the hybridisation of DNA to 50-mer oligonucleotide sequences. The presence of a high degree of paralogy within the *Malus* genome could lead to non-preferential binding of paralogous gene sequences to what were thought to be locus-specific probes on the IRSC array, causing shifts in clustering patterns obtained from SNP genotyping, and the erroneous scoring of segregating loci in the investigation of Antanaviciute et al [20]. Furthermore, since the parents of the M432 progeny represent divergent germplasm in relation to the genotypes used to develop the IRSC array, additional segregating SNP loci present in the probe binding sites may have altered binding efficiency of probes, that could generate unexpected segregation types, and in extreme cases null alleles, as has been observed previously when scoring grapevine (*V. vinifera*) SNPs using other genotyping technology [21].

In this investigation, we re-evaluated data generated using the IRSC array for an apple rootstock progeny. We identified widespread incidences of incorrectly assigned genotypes following automatic genotype calling, in both the mapping progeny, and in data from a selection of 192 apple varieties. We attribute the incorrectly assigned genotypes to probe binding sequence paralogy and divergence. The data we present in this analysis demonstrate the potential difficulties that can be encountered when using high-throughput microarray-based genotyping applications in complex, heterozygous, genomes such as apple, in which recent WGD have occurred, but also in polyploid species.

## Materials and Methods

### BLAST Analysis of the ‘Golden Delicious’ Genome Sequence using IRSC Probes as Queries

To determine the proportion of probes that could potentially anneal to paralogous regions of the *Malus* genome, probe sequences from the IRSC array were used as queries to BLAST the ‘Golden Delicious’ genome sequence at two different levels of stringency. Only regions that returned a perfect match between the 50 nucleotides of the probes and the genome sequence were considered as positive for the first BLAST analysis, following the Illumina guidelines for probe development. Subsequently, 24 nucleotides from the 3′ end of the probe were used as queries, since this is the section of the probe sited closest to the SNP under assay and thus the most critical section in which to conserve sequence integrity. Due to the physical-chemical properties of DNA oligonucleotide binding and tolerances permitted in nucleotide divergence [22] up to two mismatches between the probe and the genome sequence were permitted for a result to be considered positive.

### Manual Inspection and Annotation of SNP Loci

Data for the IRSC array generated previously by Antanaviciute et al [20] for the M432 apple rootstock mapping progeny were used in this investigation. Data were clustered using GenomeStudio (Illumina) and all 8,788 loci were scrutinised visually. Loci called incorrectly by the automated genotype calling of GenomeStudio (Illumina) were manually annotated. The 8,788 SNP probes on the IRSC array were genotyped in a collection of 192 diverse apple (*Malus pumila*) (Table S1) varieties following the standard Illumina protocol detailed in [20]. Automatic clustering using GenomeStudio (Illumina) was followed by visual inspection of data for the heterozygous probes mapped in the M432 mapping progeny and assigned genotype clusters were evaluated as described above for the M432 progeny.

### Comparison of Paralogous Sequence Variants with Genotype Cluster Positions

Single copy, heterozygous SNPs segregating in a diploid genome, cluster with mean normalised Theta values approximating to 0 for an *AA* genotype, 1 for a *BB* genotype and 0.5 for an *AB* genotype. Data generated for SNPs heterozygous in the M432 progeny were exported in text format from GenomeStudio (Illumina). A custom Python script was written to hierarchically cluster these data with a simple agglomerative strategy based on the Euclidian distance of the normalised Theta and normalised R coordinates for each genotype at each locus. The Python script is available upon request from the corresponding author. Several clustering runs were performed with similarity thresholds between clusters of 0.1, 0.15, 0.2, 0.3, 0.4 and 0.5. Clusters composed of fewer than three individuals were removed. The mean normalised Theta and R values for all markers that segregated in a 1∶1 or 1∶2:1 manner at a similarity threshold of 0.15, and for which BLAST data were available, were plotted according to whether the probe sequences returned a single BLAST hit to the ‘Golden Delicious’ genome, or multiple significant matches on multiple chromosomes, and the plots were compared.

### SNP Co-segregation Analysis and Integration into the M432 Consensus Linkage Map

Manually annotated markers were integrated into the consensus linkage map of Antanaviciute et al [20] using JOINMAP 4.0 (Kyasma, Wageningen, NL) keeping the marker order of previously mapped SNPs fixed as the ordering of SNPs on that map had been previously demonstrated to be reliable. Marker placement was determined using a minimum LOD score threshold of 5.0, a recombination fraction threshold of 0.35, ripple value of 1.0, jump threshold of 3.0 and a triplet threshold of 5.0, and the Kosambi mapping function. The genetic positions of the newly mapped SNPs were plotted using MAPCHART 2.2 for Windows [23].

## Results

### BLAST Analysis of the ‘Golden Delicious’ Genome Sequence using IRSC Probes as Queries

**Figure 1:**
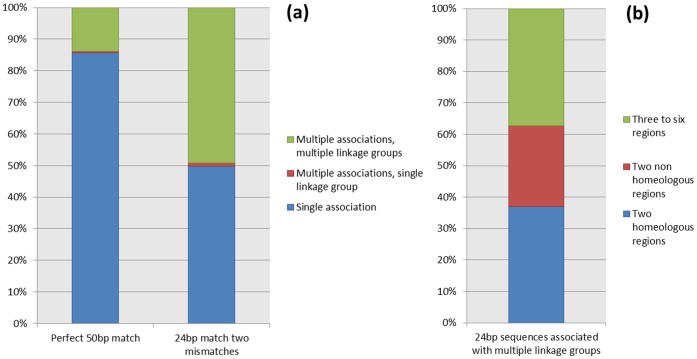
SNP probe associations on the ‘Golden Delicious’ genome. (a) The percentage of probes associated with single loci, multiple loci on a single linkage group and multiple loci on multiple linkage groups in the ‘Golden Delicious’ genome, using perfect 50 bp probe matches and 24 bp probe matches permitting up to two mismatches in the locus sequence. (b) The percentage of 24bp probe sequences matching multiple linkage groups that were associated with two homeologous regions, two non-homeologous regions and three to six regions of the ‘Golden Delicious’ genome sequence.

Of the 8,788 probes on the IRSC SNP array, 7,901 (90%) returned perfect matches to the ‘Golden Delicious’ genome sequence assembly ‘all contigs’ set when the full 50 nucleotides of each probe were used as queries; 6,767 (85.6%) of them returned a single significant association with the ‘Golden Delicious’ genome sequence, 44 (0.6%) returned multiple hits to a single linkage group, suggesting paralogous loci that may have arisen through copy-number variation (CNV) and 1,090 (13.8%) returned multiple hits to multiple linkage groups suggesting annealing to paralogous sequences (Figure 1a). When the 24 nucleotides at the 3′ end of the probes were used as queries, the number of sequences returning hits to a single region of the ‘Golden Delicious’ genome sequence decreased to 3,931 (49.8%), the number of sequences returning hits to a single linkage group but multiple scaffolds increased to 86 (1%), whilst the number of sequences returning hits to multiple linkage groups increased to 3,884 (49.2%) (Figure 1a). Of the probes that mapped to multiple genome regions when the first 24 nucleotides from the 3′ region of the probe were used as queries for BLAST, 2,440 mapped to two genomic regions, of which 1,436 mapped to homeologous regions, whilst the remaining 1,444 mapped to between three and six chromosomes each (Figure 1b).

### Manual Annotation of SNP Loci

Cluster data generated for the M432 mapping progeny using the IRSC array by Antanaviciute et al [20] were visually re-inspected. There were a total of 942 SNPs that displayed reliable clusters, but for which segregation had been mis-scored, or a no-score had been assigned to a segregating SNP by GenomeStudio (Illumina). Of the 942 loci identified, 870 were derived from *Malus* SNP assays and the remaining 72 were derived from *Pyrus* SNP assays.

**Figure 2:**
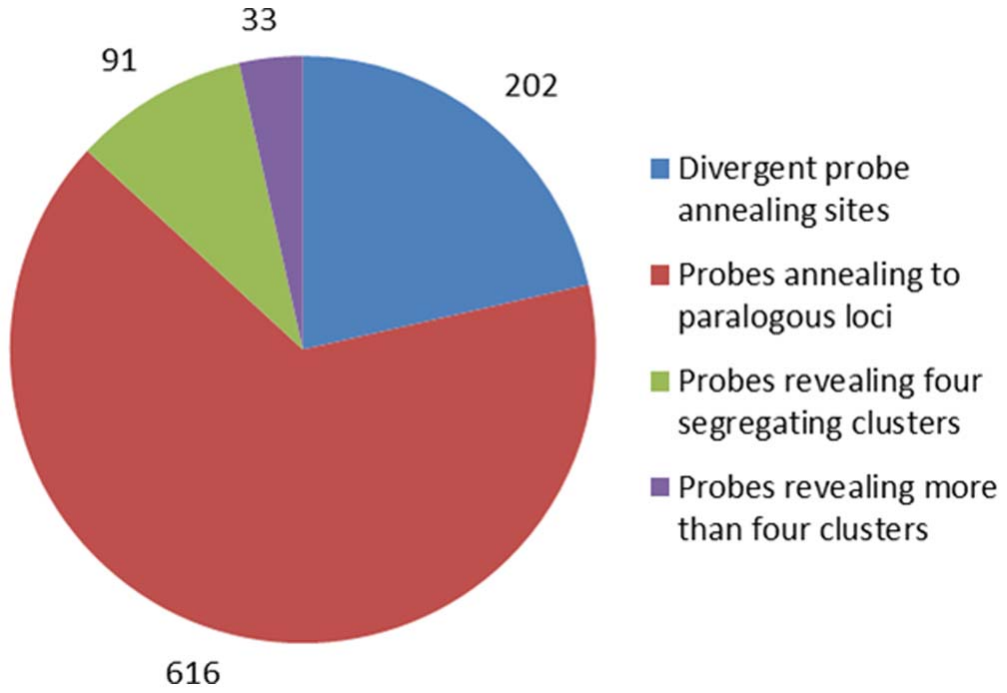
Summary of SNP markers reclassified through manual annotation. Reclassification of the 921 probes manually annotated in this investigation into probes segregating in two or three clusters with divergent probe annealing sites (*n* = 202) or annealing to paralogous loci (*n* = 616), and those revealing four segregating clusters (*n* = 91), or more than four clusters (*n* = 33).

Each of the misclassified loci for which clusters could be reliably differentiated, was manually annotated and each of the seedlings in the population was genotyped at each locus. Markers previously classified as monomorphic (i.e. AA×AA or BB×BB), and a number of those classified previously as segregating in a 1∶1 ratio (i.e. AA×AB or AB×BB) were reclassified variously as segregating either 1∶1 or 1∶2:1 for those displaying evidence of binding to paralogous sequences (Figure S1), and 1∶1, 3∶1 and 1∶2:1 for those displaying evidence of reduced probe binding efficiency due to the presence of additional SNPs in the probe binding sites (Figure S2). SNPs were only called at loci where clear unambiguous separation of clusters was observed. A total of 818 loci could be reliably annotated and assigned either to two or three genotype classes. Of these, 745 were derived from *Malus* SNP assays and the remaining 73 were derived from *Pyrus* SNP assays. Of the remaining 124 loci, 91 loci produced four clear clusters of genotypes, suggesting the segregation of three alleles at the locus (i.e. AB×AC) which could not be assigned genotypic classes for mapping, whilst 33 loci were not annotated because the number of genotype classes observed after cluster separation exceeded four, suggesting segregation of more than one heterozygous locus (i.e. ABAB×ABAB). Figure 2 summarises how each of the 942 loci were reclassified in this investigation.

### SNP Co-segregation Analysis and Integration into the M432 Consensus Linkage Map

A total of 469 SNPs segregated approximating to a 1∶1 Mendelian ratio, 235 in the female ‘M.27′ genotype, 234 in the male ‘M.116′ genotype, whilst 68 and 281 SNPs segregated approximating to a 3∶1 and a 1∶2:1 Mendelian ratio respectively. Following co-segregation analysis with the data of Antanaviciute et al [20], 797 SNPs (725 from *Malus* and 72 from *Pyrus*), 449 segregating approximating to a 1∶1 segregation ratio, 280 segregating approximating to a 1∶2:1 ratio and 68 segregating approximating to a 3∶1 ratio, mapped to one of the 17 linkage groups of the M432 map, bringing the total number of SNP markers mapped using the IRSC array in the M432 progeny to 3,069. The remaining 21 loci, all of which displayed cluster patterns suggesting annealing to paralogous loci, remained unlinked. The manually-annotated SNPs mapped across the 17 linkage groups of the M432 linkage map, and no clustering of re-scored loci was observed suggesting genome-wide incidences of probes binding to paralogous loci and reduced probe binding efficiency due to probe sequence divergence. All markers exhibiting recombination from previously mapped markers, and thus unique genotypes, were visually scrutinised to ensure no spurious genotyping calls had been made following which, a total of just 15.2% of the mapped SNPs revealed unique genotypes and thus map positions. The additional loci did not extend the M432 linkage map significantly, suggesting data for loci mapped in this investigation are robust and reliable. Figure S3 shows the genetic positions of the 797 SNP markers whilst Table S2 details their marker names, segregation ratios and physical positions on the ‘Golden Delicious’ genome sequence.

### Physical vs. Genetic Positions on the M432 Linkage Map

**Figure 3:**
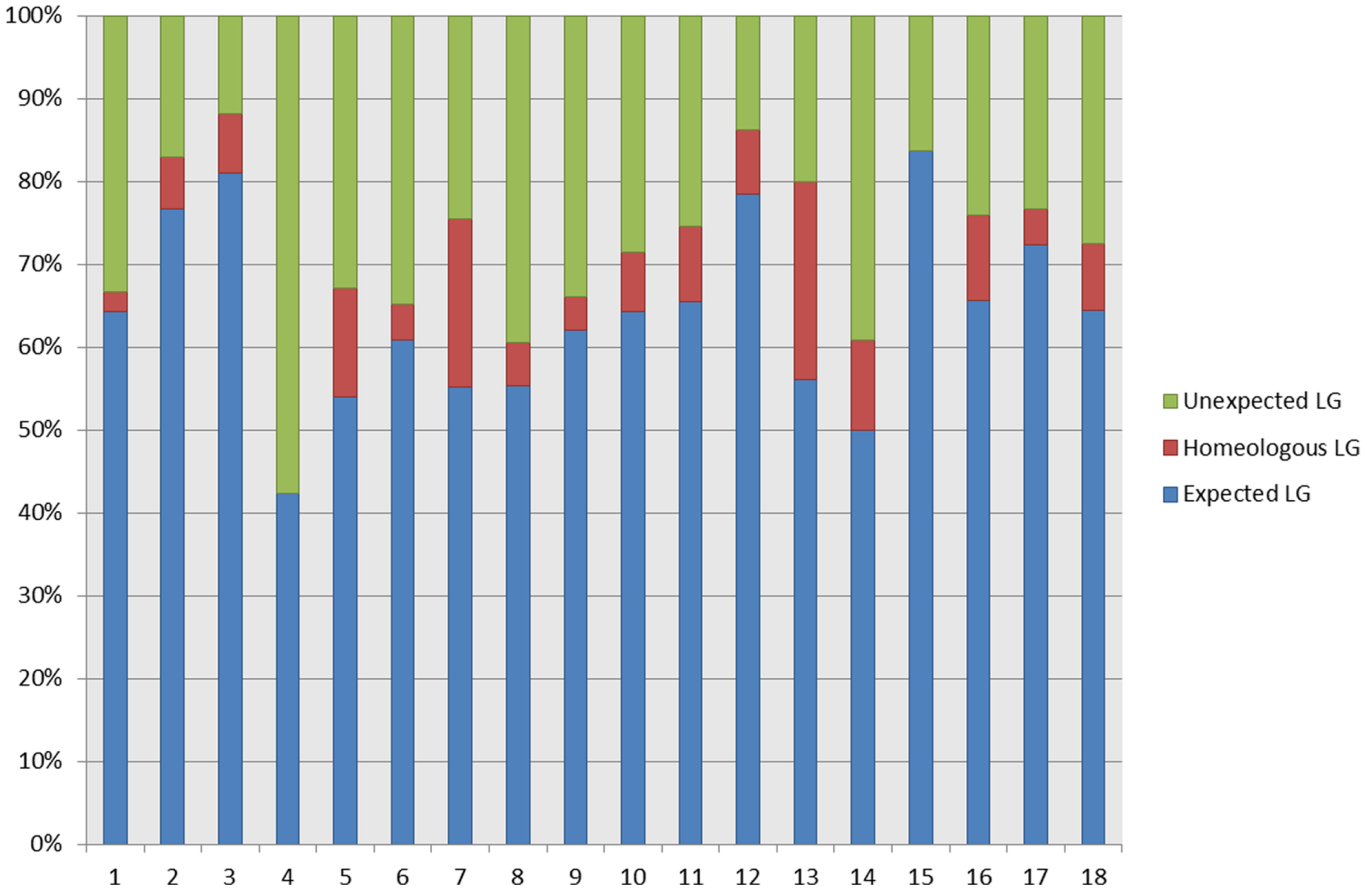
A comparison of genetic and physical positions of probes mapped in M432. A comparison of the percentage of probes mapped to each linkage group on the M432 linkage map with their physical positions on the ‘Golden Delicious’ genome sequence.

Of the 797 markers mapped in this investigation, 67 were not physically located to the ‘Golden Delicious’ genome. Of the remaining 730 markers, 470 (64.4%) mapped to the expected linkage group based on the physical location of the marker on the ‘Golden Delicious’ genome, 59 (8.1%) mapped to the identified homeologous linkage group [3] to the pseudochromosome on which the marker was physically positioned, and the remaining 201 (27.5%) markers mapped to an unexpected linkage group based on physical location. The proportion of markers mapping to expected linkage groups was not even and varied depending on which linkage group the marker mapped to. Linkage group 4 contained the highest proportion of novel markers that were expected to map alternative LGs based on marker physical positions, whilst LG3 contained the highest proportion of markers that were located on the expected group based on physical marker positions. Figure 3 displays the proportions of markers mapped to each of the 17 linkage groups of the M432 linkage map that were located on expected, homeologous or unexpected pseudochromosomes.

### Comparison of BLAST Analysis and Genotype Clustering and SNP Transferability

**Figure 4:**
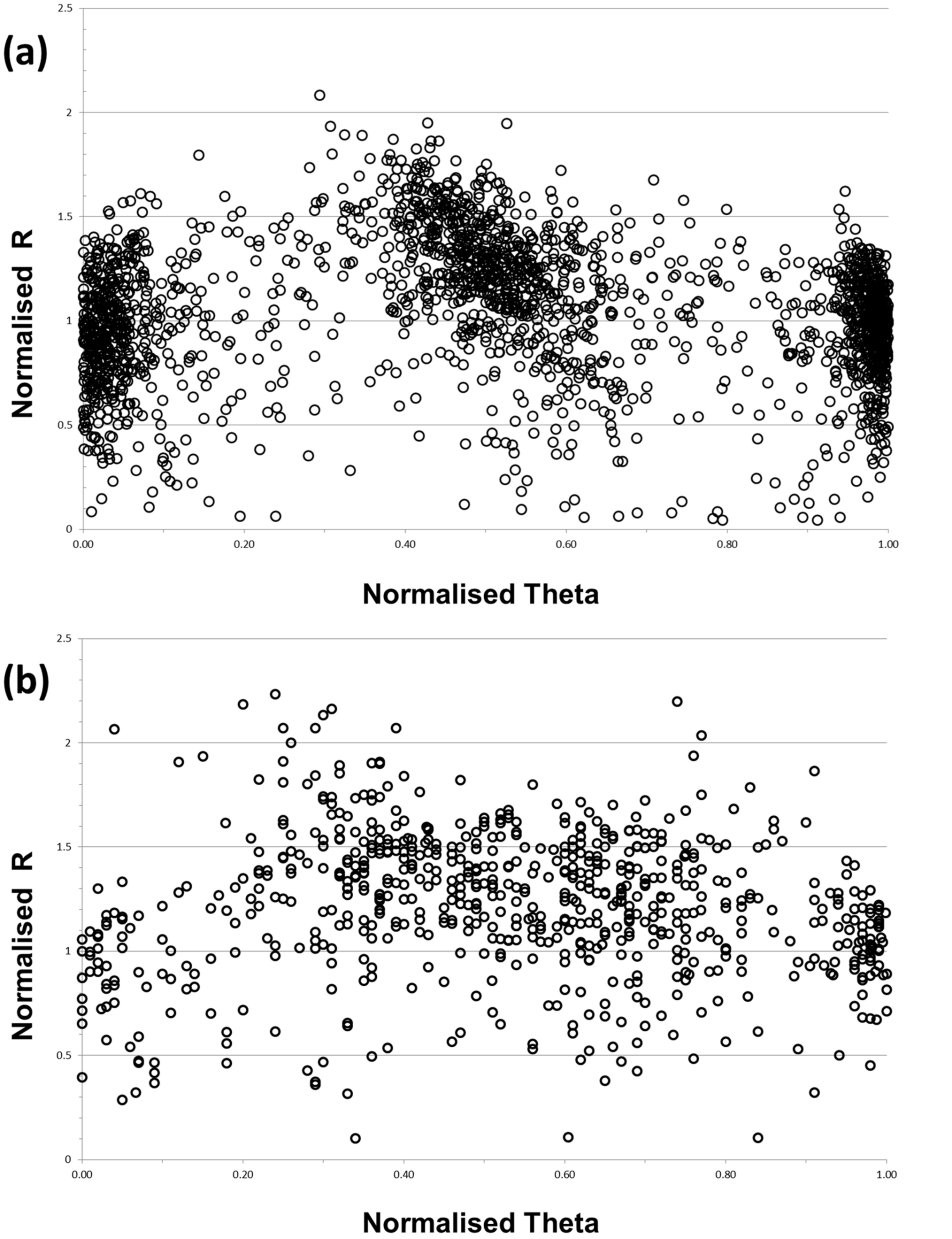
Cluster plots of IRSC probes. Mean normalised Theta and normalised R values from markers genotyped in the M432 progeny using the IRSC array plotted for (a) probes to which a single significant genome match was returned and (b) probes returning multiple hits to the ‘Golden Delicious’ genome sequence.

BLAST and cluster analysis data were available for a total of 1,387 heterozygous marker clusters, of which 468 returned significant hits to multiple chromosomes in the ‘Golden Delicious’ genome sequence, whilst the remaining 919 returned a significant match to a single position. When the mean Theta and R values were plotted for each of these loci, clear differences were observed regarding the position of the clusters within graph space (Figure 4), with the probes to which a single significant genome match was returned (Figure 4a), clustering in general close to the expected positions at 0, 0.5 and 1 on the graph, whilst the clustering of probes returning multiple hits to the ‘Golden Delicious’ genome sequence (Figure 4b) were far more widely distributed across graph space, indicating that probe sequence paralogy was responsible for the unexpected distribution of genotype clusters in graph space.

**Figure 5:**
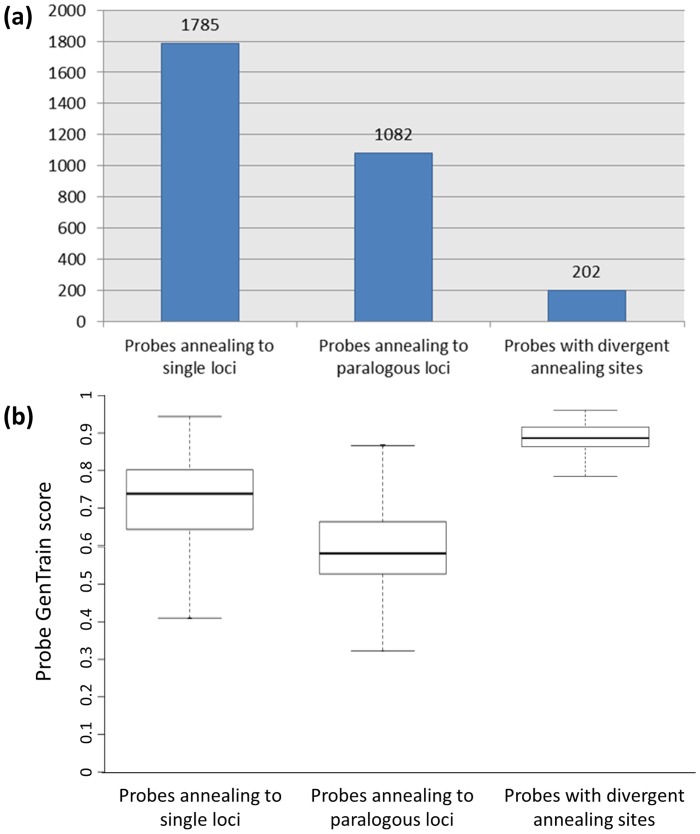
Comparison of probe annealing numbers, behaviour and probe GenTrain scores. (a) The number of probes mapped in the M432 progeny showing evidence of annealing to single loci, multiple paralogous loci and probes showing evidence of probe annealing site divergence. (b) Box and whisker plots comparing the GenTrain scores of probes associated with single loci, and with multiple loci in the ‘Golden Delicious’ genome following BLAST analysis, and probes for which divergent annealing sites contained additional segregating SNPs or created null alleles.

Figure 5a shows the numbers of markers mapped in the M432 progeny displaying expected cluster separation profiles, profiles showing evidence of probe sequence paralogy and profiles showing evidence of additional segregating SNPs in probe annealing sites or null alleles. Of the 797 markers mapped for the first time in M432 in this investigation, 202 were called by GenomeStudio (Illumina) in the previous investigation of Antanaviciute et al [20] as monomorphic but were shown to display horizontal shifts in genotype clusters following visual inspection and rescoring. These genotype clusters had the same normalised Theta values, but significantly different normalised R values indicating a lower efficiency in probe binding for one of the clusters due to divergence in the probe binding sequence within the genome of the M432 parental varieties. For 106 of these loci, the normalised R value of one of the clusters observed approached, or was equal to 0, indicating a complete lack of probe binding, and the cluster was called as a null allele (Figure S2). The genotype clusters for the remaining 595 loci displayed deviations from the expected normalised Theta values of 0, 0.5 and 1 leading to mis-scored and un-scored data using automatic genotype calling in the previous investigation of Antanaviciute et al [20]. Manual re-evaluation and scoring of these loci suggested the errors were caused by binding to paralogous loci (Figure S1). Furthermore, 487 (21.5%) of the 2,272 mapped loci that were called correctly using automated genotype calling by GenomeStudio (Illumina) [20] showed evidence of probes binding to paralogous loci and thus deviation from the expected clustering positions on in graph space.

Of the newly mapped SNPs derived from the 7,867 *Malus* probes IRSC array, a total of 157 (19.7%) were derived from suspected divergence in the probe sequence binding site in the M432 parents, 92 of which created a null allele, whilst the remaining 568 (80.3%) SNPs annealed to paralogous loci in the *Malus* genome. In contrast, 45 (63.4%) SNPs derived from the 921 *Pyrus* probes on the IRSC array segregated due to heterozygosity due to suspected divergence in the probe sequence binding site in the M432 parents, 14 of which created a null allele, whilst just 27 (36.6%) annealed to paralogous loci (Table 1) indicating probe binding efficiency reduces with increasing genetic distance between the genotypes used for probe development and genotyping.

**Table 1:** The number of SNP markers derived from both *Malus* and *Pyrus* genomic sequence mapped in this investigation displaying evidence for probe sequence variants, null alleles and non-specific probe binding.

Cluster types	Marker segregation	Number of SNPs	Percentage of total number of SNPs
*Malus*			
Mutation in probe site but SNP amplified	1∶1	51	7.0
	1∶2:1	6	0.8
	3∶1	8	1.1
Mutation in probe site null allele	1∶1	59	8.1
	1∶2:1	27	3.7
	3∶1	6	0.8
Probe sequence binding paralogous loci	1∶1	295	40.70
	1∶2:1	229	31.60
	3∶1	44	6.2
**Total**		**725**	
*Pyrus*			
Mutation in probe site but SNP amplified	1∶1	24	33.3
	1∶2:1	2	2.8
	3∶1	5	6.9
Mutation in probe site null allele	1∶1	11	15.3
	1∶2:1	1	1.4
	3∶1	2	2.8
Probe sequence binding paralogous loci	1∶1	11	15.3
	1∶2:1	13	18.1
	3∶1	3	4.1
**Total**		**72**	

Probes that BLAST data suggested annealed to a single genomic locus had slightly better GenTrain quality scores than those which BLAST suggested annealed to paralogous loci. However, probes for which annealing sites were divergent, and thus for which additional segregating SNPs were present in the probe sites, or for which null alleles were observed had significantly better GenTrain quality scores than either of the other two classes of probe (Figure 5b).

### Manual Evaluation of Genotype Clustering in a Malus Pumila Cultivar Collection

**Figure 6:**
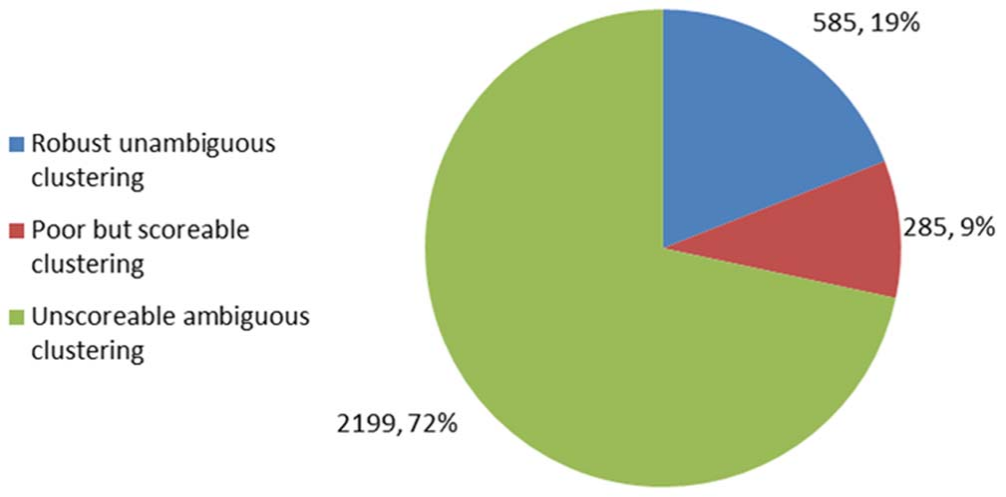
Summary of manual annotation of mapped probes genotyped in a *Malus* germplasm collection. The number of probes displaying either robust, reliable genotypes; ambiguous but scoreable genotypes; and unscoreable genotypes following genotyping of a collection of 192 *Malus* cultivars.

Manual annotation of the 192 cultivars revealed a total of 585 of the 3,069 markers mapped (19%) in the M432 progeny that displayed robust, reliable genotypes containing no null alleles and clustering in one, two or three distinct genotype groups. A further 285 (9%) probes displayed a low level of ambiguity that may have been the result of too few genotypes in a particular class and could have revealed clearer clustering with the inclusion of further cultivars in the analysis. The remaining 2,199 (72%) probes revealed cluster patterns containing more than three clusters and could not be assigned to a genotype Figure 6.

## Discussion

Genotyping using high-throughput microarray technology has the potential to fundamentally change the way genomes are genetically characterised and has vastly increased the level of resolution at which genetic analyses are performed. High and ultra-high throughput mammalian Infinium arrays are available for genotyping cows, dogs, pigs, sheep, and goats, and high-throughput Infinium arrays available for maize, peach [15], cherry, tomato and grapevine [13] have been used to successfully genotype both mapping progenies and germplasm collections. However, the data we present here suggest, as has been discussed recently in in a review of genomics resources for grapevine [24], that in organisms with complex heterozygous genomes, especially those such as *Malus* in which WGD events have occurred relatively recently, high degrees of paralogy in the genome and high levels of sequence divergence between genotypes within probe sequences will render the interpretation of a large proportion of the data generated from genotyping arrays problematic if they have not been taken into consideration during the initial design of the array.

Previously, using automatic genotype calling with GenomeStudio (Illumina), Antanaviciute et al [20] developed a linkage map for the M432 mapping population containing a total of 2,272 SNP loci mapped using segregation data generated using the IRSC array [19]. However, an additional 586 segregating loci could not be mapped due to spurious automatic genotype calls as a result of probes binding to paralogous regions of the genome. In this investigation we re-evaluated the data generated using the 8,788 SNP assays contained on the IRSC array and by manually assigning genotypes to markers assigned a ‘no-call’ or erroneously scored using automatic genotype calling with GenomeStudio (Illumina), and placed a further 797 heterozygous SNP loci to the M432 linkage map. These newly-mapped loci included the majority of the 586 markers that were not mapped by Antanaviciute et al [20] as well as loci that had previously been scored as monomorphic but which segregated due to probe sequence divergence leading to different binding efficiencies of the two segregating alleles. The addition of these loci increased the number of mapped SNPs by 35% and reduced the number of heterozygous loci that could not be mapped to just 21.

The genus *Malus* to which the cultivated apple belongs is a member of the Pyreae tribe of the Spiraeoideae subfamily of the Rosaceae [25], which comprises other tree-fruit genera including pears (*Pyrus* spp.), quince (*Cydonia oblonga* Mill.) and loquat (*Eriobotrya japonica* Lindl.) and ornamental genera such as the rowans (*Sorbus* spp.), all of which have the basic diploid genome configuration 2*n* = 2*x* = 17. However, the 17 chromosomes of the Pyreae tribe are derived from a recent WGD event as was demonstrated recently by Velasco et al [3] following the sequencing of the ‘Golden Delicious’ genome, who showed clear homeologous relationships between *Malus* LGs 5 and 10; 3 and 11; 9 and 17; 13 and 16; 6 and 14, and more complex relationships between 4, 12 and 14; 1, 7 and 2; and 8 and 15. As a result of this recent duplication, the *Malus* genome contains the largest reported number of genes of any plant genome sequenced to date and as such, an exceptionally high number of paralogous loci have been identified in the sequence. Besides the more recent WGD, sequencing of the ‘Golden Delicious’ genome sequence also demonstrated the presence of other more ancient duplication events, including the ancient hexaploidisation event in the lineage of the angiosperms [3].

### Identification of Paralogous Sequence Variation

In line with the findings of Velasco et al. [3], the majority of the probes that revealed multiple paralogous annealing sites in the *Malus* genome sequence aligned to two distinct regions, indicative of the WGD event that occurred 30–45 Mya in the evolution of the Pyrea tribe [3]. However, approximately one quarter (27.5%) of the total mapped probes aligned to non-homeologous genomic regions, higher than the 13.7% of probes identified by Antanaviciute et al. [20], highlighting evidence of either extensive inter-chromosomal rearrangements which occurred in the *Malus* genome following the WGD event, or possible inconsistencies in the ‘Golden Delicious’ genome sequence assembly. A minority of probe sequences returned significant alignments to between three and six *Malus* linkage groups, consistent with the paleohexaploidisation event shown to have occurred in the evolutionary lineage of the angiosperms, prior to the WGD event in the Pyrea tribe [26], [27].

The recent WGD event, more ancient polyploidisation events, and instances of expansion of gene families have all contributed to the high degree of paralogy observed within the *Malus* genome. BLAST analysis at two levels of stringency of the 8,788 probes contained on the IRSC array clearly demonstrated the potential for paralogy to lead to non-specific annealing of probes within the *Malus* genome. This was evidenced by a three-fold increase in probe annealing site paralogy when the criteria for the BLAST analysis were changed to evaluate just the first 24 nucleotides of probe sequences. This study identified erroneously scored data for a total of 595 SNP loci due to shifts in genotyping cluster positions caused by the binding of probe sequences to paralogous loci within the *Malus* genome. However, a further 487 probe sequences successfully scored using the automated genotype calling function of GenomeStudio (Illumina) and previously mapped by Antanaviciute et al [20] displayed evidence of annealing to paralogous loci equating to evidence of paralogy for 35% of all loci mapped in the M432 progeny. GenTrain scores were within similar ranges for probes associated with a single locus in the ‘Golden Delicious’ genome and those associated with multiple loci, suggesting such metrics cannot be used to reliably predict probe performances in genetic analyses. Surprisingly however, those probes which displayed evidence of divergent annealing sites containing multiple segregating SNPs or creating segregating null alleles returned significantly better GenTrain scores than the other two classes of probe, suggesting that the algorithm in GenomeStudio (Illumina) that derives the GenTrain score places more weight on shifts in normalised Theta between clusters than in normalised R.

Gene duplication and thus the occurrence of paralogous loci within a genome is a phenomenon that occurs in all eukaryotic genomes [28]. In the case of species with genomes that have undergone recent WGD events, it is widespread throughout much, if not all of the genome of the organism, depending on the length of time that has passed since polyploidisation. Such paralogy has been reported to have a significant effect on the efficacy of high throughput WGG array genotyping. In mammalian species, in excess of 99% of markers contained on bovine and ovine arrays were scored robustly and reliably in numerous individuals [29], [30]. Peach is a diploid tree-fruit species, and the peach 9k Infinium array [15] was reported to contain a total of 6,989 SNPs polymorphic in 709 peach accessions, but no difficulties in scoring the loci due to paralogy or probe annealing sequence divergence was reported. Likewise, the *Vitis* 9k array [13] was not reported to contain SNPs that could not be scored due to annealing to paralogous loci or inefficient probe annealing, when screening diploid grape cultivars, however the authors augmented the potential for development of probes that detected paralogous loci by rejecting any SNPs during the process of array development that were shown to have greater than 1,000 fold coverage following re-sequencing, and thus displayed evidence for paralogy.

In contrast, in the polyploid species oilseed rape (*Brassica napus* L.), SNP assays contained on a WGG array developed using GoldenGate technology were demonstrated to have amplified two paralogous loci, both of which were heterozygous [31]. However, the authors did not quantify how many probes detected multiple loci. The maize genome underwent an ancient WGD, followed by diploidisation [32], and the Infinium array developed for maize was reported to contain a total of 6,525 SNP markers (11.6%) that displayed evidence of un-compact genotype clustering, clusters shifted due to the detection of paralogous loci, or more than the expected number of clusters due to segregation of at least two heterozygous paralogous loci. Maize, like apple, is a highly diverse species, with SNPs reported every 44–75bp throughout the genome [33]. However the data presented herein suggests that the paralogy and probe sequence divergence within the *Malus* genome has affected significantly more loci contained on the IRSC array than other polyploid species for which Infinium arrays are available.

WGG arrays have enormous potential to facilitate the execution of genome-wide association studies (GWAS). However, for such analyses to be successful, it is vital that reliable genotypic data can be generated for many tens of thousands of loci, particularly in species such as *Malus* where linkage disequilibrium decay is high [7]. Thus for the development of WGG arrays containing many tens or hundreds of thousands of SNPs for species with genomes with high proportions of paralogous loci, we have demonstrated that it is extremely important to judiciously select probe sequences to minimise the degree of paralogous probe annealing and thus the incidence of data that is difficult to interpret reliably. This could be achieved through the re-sequencing of a large number of genotypes encompassing as much of the variation that exists within commercial cultivars and breeding lines as possible. Efforts should also be taken to identify potential paralogous probe annealing sites during the development pipeline, as was done previously for grapevine [13]. This is particularly so because manual annotation of genotypic data from even tens of thousands of loci is at best unfeasible.

### IRSC Probe Transferability and Mapping

Mismatches between the IRSC probe sequences and the DNA of the progeny from the M432 mapping population, due to the presence of InDels and additional SNPs, interfered with hybridization signals and caused the failure of a hybridisation assay due to reduced hybridisation efficiency between the probe and the DNA being assayed. This phenomenon is not unique to apple and has been shown to occur in other crop species such as maize and grape [13], [14]. In fact, such variation is common in genomes of high diversity species such as *Malus*, and in maize, the rate of failure has been strongly correlated to the genetic distance of the genotype being assayed from the maize reference genome [14]. However, segregation for such variation if alleles are present in a heterozygous state can be scored, and loci can be mapped using data for null alleles or for additional SNPs. In this investigation, we have demonstrated through manual annotation that such variation caused separation in the clustering along the vertical (normalised R) axis of the cluster space in GenomeStudio (Illumina), which was not detected through automatic genotype calling.

Micheletti et al. [7] reported a transferability rate of heterozygous SNPs (T_SNP_) from *Malus* to *Pyrus* of between 1.2 and 3.0% for *P. pyrifolia* and *P. communis* respectively using the SNPlex platform. Likewise Antanaviciute et al [20] reported a transferability rate of 2.8% (26 SNPs) of heterozygous *Pyrus* SNPs to the M432 progeny when automatic SNP genotype calling was used. In this investigation, we demonstrated that manual annotation of genotype clusters produced from probes developed from *Pyrus* genomic sequences recovered a far higher percentage of heterozygous loci in *Malus* germplasm than would have been expected from T_SNP_ values reported for transferability from *Pyrus* to *Malus*. This was due to the presence of other polymorphisms associated with the probe sequence and the existence of such variation has implications for the use of these probes in GWAS.

### Conclusions

The development of array-based genotyping for *Malus* provides numerous experimental advantages over genotyping using traditional PCR-based assays analysed by electrophoresis [34]. However, the detection of high levels of paralogous annealing sites and annealing site divergence in probes contained on the IRSC array has implications for the future development of arrays for species as divergent and heterozygous as *Malus* since both phenomena, especially when they occur together at a locus, make genotyping data difficult to score reliably. This has been demonstrated in this study through the genotyping of a set of *Malus* varieties from a diverse germplasm collection, where the majority of loci scored displayed evidence of these phenomena and rendered genotyping unreliable or impossible. For the development of a widely applicable tool for genotyping and marker-trait association, it is thus important to consider the degree of sequence divergence within the probe sequences by developing probes with the assistance of the largest re-sequencing resource possible, encompassing as diverse a germplasm base as possible. This will minimise the level of undetected probe sequence divergence, and thus the prevalence of null alleles and additional multi-SNP alleles within genotyping datasets.

## Supporting Information

Figure S1 **Erroneously scored genotype clusters annealing to multiple genomic regions.** Examples of erroneously scored genotype clusters following automatic genotyping using GenomeStudio (Illumina)of SNP that target multiple sites. Red circles and genotypes written in red indicate genotype assignment by GenomeStudio: (a) Data scored as monomorphic AA clearly clustering in three genotype groups (AA/AA:AB/AA:BB/AA); (b) data scored as segregating 1∶1 (AA:AB) clearly segregating in three genotype groups (AA/AA:AB/AA:BB/AA); (c) Data scored as monomorphic AA clearly segregating in two genotype groups (AA/AA:AB/AA); (d) Data scored as monomorphic BB clearly clustering in three genotype groups (AA/BB:AB/BB:BB/BB); (e) data scored as segregating 1∶1 (AB:BB) clearly segregating in three genotype groups (AA/BB:AB/BB:BB/BB); (f) Data scored as monomorphic BB clearly segregating in two genotype groups (AB/BB:BB/BB).
(PNG)

Figure S2 **Erroneously scored genotype clusters containing additional SNPs.** Examples of erroneously scored genotype clusters following automatic genotyping using GenomeStudio (Illumina) containing additional SNPs (hereafter reported as B’) and null (n) alleles. Red circles and genotypes indicate scores assigned by GenomeStudio, blue circles indicate genotypes assigned ‘no call’ (NC) by GenomeStudio: (a) Data scored as monomorphic BB clustering in two genotype groups (BB’:BB) resulting from BB’×BB; (b) Data scored as BB clustering in three genotype groups (BB:BB’:B’B’) resulting from BB’×BB’; (c) Data scored as BB clustering in two genotype groups (BB or BB’:B’B’) resulting from BB’×BB’; (d) Data scored as BB clustering in two genotype groups (Bn:nn) resulting from Bn×nn; (e) Data scored as BB clustering in three genotype groups (BB:Bn:nn) resulting from Bn×Bn; (f) Data scored as BB clustering in two genotype groups (BB or Bn:nn) resulting from Bn×Bn.
(PNG)

Figure S3 **Linkage map of newly-mapped SNP markers.** The M432 consensus linkage map detailing the map positions of the 797 novel SNP loci mapped in this investigation following manual re-annotation of data generated with the IRSC array. The scale in centi-Morgans (cM) is given at the left edge of the figure.
(PNG)

Table S1 **The names of the 192** ***Malus*** **varieties genotyped with the IRSC array.**
(PDF)

Table S2 **The genetic positions, marker names, segregation ratios and physical positions on the ‘Golden Delicious’ genome sequence of the 797 SNP markers mapped to the M432 linkage map.**
(XLS)

## References

[1] Velasco R, Zharkikh A, Troggio M, Cartwright DA, Cestaro A, et al (2007) A high quality draft consensus sequence of the genome of a heterozygous grapevine variety. PLoS One 2: e1326. 18094749 10.1371/journal.pone.0001326 PMC2147077

[2] Ward JA, Bhangoo J, Fernández-Fernández F, Moore P, Swanson JD, et al (2013) Saturated linkage map construction in *Rubus idaeus* using genotyping by sequencing and genome-independent imputation. BMC Genomics 14: 2. 23324311 10.1186/1471-2164-14-2 PMC3575332

[3] Velasco R, Zharkikh A, Affourtit J, Dhingra A, Cestaro A, et al (2010) The genome of the domesticated apple (*Malus* x *domestica* Borkh.). Nat Genet 42: 833–839. 20802477 10.1038/ng.654

[4] Bus A, Hecht J, Huettel B, Reinhardt R, Stich B (2012) High-throughput polymorphism detection and genotyping in *Brassica napus* using next-generation RAD sequencing. BMC Genomics 13: 281. 22726880 10.1186/1471-2164-13-281 PMC3442993

[5] Ollitrault P, Terol J, Garcia-Lor A, Berard A, Chauveau A, et al (2012) SNP mining in C. clementina BAC end sequences; transferability in the *Citrus* genus (Rutaceae), phylogenetic inferences and perspectives for genetic mapping. BMC Genomics 13: 13. 22233093 10.1186/1471-2164-13-13 PMC3320530

[6] Scaglione D, Lanteri S, Acquadro A, Lai Z, Knapp SJ, et al (2012) Large-scale transcriptome characterization and mass discovery of SNPs in globe artichoke and its related taxa. Plant Biotechnol J 10: 956–969. 22849342 10.1111/j.1467-7652.2012.00725.x

[7] Micheletti D, Troggio M, Zharkikh A, Costa F, Malnoy M, et al (2011) Genetic diversity of the genus *Malus* and implications for linkage mapping with SNPs. Tree Genet Genomes 7: 857–868.

[8] Myles S, Boyko AR, Owens CL, Brown PJ, Grassi F, et al (2011) Genetic structure and domestication history of the grape. Proc Natl Acad Sci USA 108: 3530–3535. 21245334 10.1073/pnas.1009363108 PMC3048109

[9] Vezzulli S, Troggio M, Coppola G, Jermakow A, Cartwright D, et al (2008) A reference integrated map for cultivated grapevine (*Vitis vinifera* L.) from three crosses, based on 283 SSR and 501 SNP-based markers. Theor Appl Genet 117: 499–511. 18504538 10.1007/s00122-008-0794-3

[10] Martinez-Garcia PJ, Fresnedo-Ramirez J, Parfitt DE, Gradziel TM, Crisosto CH (2013) Effect prediction of identified SNPs linked to fruit quality and chilling injury in peach *Prunus persica* (L.) Batsch. Plant Mol Biol 81: 161–174. 23184287 10.1007/s11103-012-9989-8

[11] Riedelsheimer C, Lisec J, Czedik-Eysenberg A, Sulpice R, Flis A, et al (2012) Genome-wide association mapping of leaf metabolic profiles for dissecting complex traits in maize. Proc Natl Acad Sci USA 109: 8872–8877. 22615396 10.1073/pnas.1120813109 PMC3384205

[12] Steemers FJ, Gunderson KL (2007) Whole genome genotyping technologies on the BeadArray platform. Biotechnol J 2: 41–49. 17225249 10.1002/biot.200600213

[13] Myles S, Chia J-M, Hurwitz B, Simon C, Zhong GY, et al (2010) Rapid genomic characterization of the genus Vitis. PLoS One 5(1): e8219. 20084295 10.1371/journal.pone.0008219 PMC2805708

[14] Ganal MW, Durstewitz G, Polley A, Berard A, Buckler ES, et al (2011) A Large Maize (*Zea mays* L.) SNP genotyping array: Development and germplasm genotyping, and genetic mapping to compare with the B73 reference genome. PLoS One 6(12): e28334. 22174790 10.1371/journal.pone.0028334 PMC3234264

[15] Verde I, Bassil N, Scalabrin S, Gilmore B, Lawley CT, et al (2012) Development and evaluation of a 9K SNP Array for peach by internationally coordinated SNP detection and validation in breeding germplasm. PLoS One 7(4): e35668. 22536421 10.1371/journal.pone.0035668 PMC3334984

[16] Felcher KJ, Coombs JJ, Massa AN, Hansey CN, Hamilton JP, et al (2012) Integration of two diploid potato linkage maps with the potato genome sequence. PLoS One 7(4): e36347. 22558443 10.1371/journal.pone.0036347 PMC3338666

[17] Bachlava E, Taylor CA, Tang S, Bowers JE, Mandel JR, et al (2012) SNP Discovery and development of a high-density genotyping array for sunflower. PLoS One 7(1): e29814. 22238659 10.1371/journal.pone.0029814 PMC3251610

[18] Sim S-C, Durstewitz G, Plieske J, Wieseke R, Ganal MW, et al (2012) Development of a large SNP genotyping array and generation of high-density genetic maps in tomato. PLoS One 7(7): e40563. 22802968 10.1371/journal.pone.0040563 PMC3393668

[19] Chagne D, Crowhurst RN, Troggio M, Davey MW, Gilmore B, et al (2012) Genome-wide SNP detection, validation, and development of an 8K SNP array for apple. PLoS One 7(2): e31745. 22363718 10.1371/journal.pone.0031745 PMC3283661

[20] Antanaviciute L, Fernandez-Fernandez F, Jansen J, Banchi E, Evans KM, et al (2012) Development of a dense SNP-based linkage map of an apple rootstock progeny using the Malus Infinium whole genome genotyping array. BMC Genomics 13: 203. 22631220 10.1186/1471-2164-13-203 PMC3410780

[21] Pindo M, Vezzulli S, Coppola G, Cartwright DA, Zharkikh A, et al (2008) SNP high-throughput screening in grapevine using the SNPlexTM genotyping system. BMC Plant Biol 8: 12. 18226250 10.1186/1471-2229-8-12 PMC2268689

[22] Neidle S (2002) Nucleic Acid Structure and Recognition. New York: Oxford University Press Inc.

[23] Voorrips RE (2002) MapChart: Software for the graphical presentation of linkage maps and QTLs. J Heredity 93: 77–78. 10.1093/jhered/93.1.77 12011185

[24] Myles S (2013) Improving fruit and wine: what does genomics have to offer? Trends Genet 29 190–196. 10.1016/j.tig.2013.01.006 23428114

[25] Potter D, Eriksson T, Evans RC, Oh S, Smedmark JEE, et al (2007) Phylogeny and classification of Rosaceae. Plant Systemat Evol 266: 5–43.

[26] Jung S, Cestaro A, Troggio M, Main D, Zheng P, et al (2012) Whole genome comparisons of Fragaria, Prunus and Malus reveal different modes of evolution between Rosaceous subfamilies. BMC Genomics 13: 129. 22475018 10.1186/1471-2164-13-129 PMC3368713

[27] Jaillon O, Aury JM, Noel B, Policriti A, Clepet C, et al (2007) The grapevine genome sequence suggests ancestral hexaploidization in major angiosperm phyla. Nature 449: 463–465. 17721507 10.1038/nature06148

[28] Force A, Lynch M, Postlethwait J (1999) Preservation of duplicate genes by subfunctionalization. American Zoologist 39(5): 460.

[29] Matukumalli LK, Lawley CT, Schnabel RD, Taylor JF, Allan MF, et al (2009) Development and characterization of a high density SNP genotyping assay for cattle. PLoS One 4(4): e5350. 19390634 10.1371/journal.pone.0005350 PMC2669730

[30] Ramos AM, Crooijmans RPMA, Affara NA, Amaral AJ, Archibald AL, et al (2009) Design of a high density SNP genotyping assay in the pig ssing SNPs identified and characterized by next generation sequencing technology. PLoS One 4(8): e6524. 19654876 10.1371/journal.pone.0006524 PMC2716536

[31] Durstewitz G, Polley A, Plieske J, Luerssen H, Graner EM, et al (2010) SNP discovery by amplicon sequencing and multiplex SNP genotyping in the allopolyploid species *Brassica napu*s. Genome 53: 948–956. 21076510 10.1139/G10-079

[32] Wei F, Coe E, Nelson W, Bharti AK, Engler F, et al (2007) Physical and genetic structure of the maize genome reflects its complex evolutionary history. PLoS Genet 3: 1254–1263. 10.1371/journal.pgen.0030123 PMC1934398 17658954

[33] Gore MA, Chia JM, Elshire RJ, Sun Q, Ersoz ES, et al (2009) A first-generation haplotype map of maize. Science 326: 1115–1117. 19965431 10.1126/science.1177837

[34] Sargent DJ, Marchese A, Simpson DW, Howad W, Fernandez-Fernandez F, et al (2009) Development of "universal" gene-specific markers from *Malus* spp. cDNA sequences, their mapping and use in synteny studies within Rosaceae. Tree Genet Genomes 5: 133–145.

