# Supplementary figures and images for: Evaluation of SNP Data from the Malus Infinium Array Identifies Challenges for Genetic Analysis of Complex Genomes of Polyploid Origin
Source: PLoS One. 2013 Jun 27;8(6):e67407. doi: 10.1371/journal.pone.0067407 (PMC3694884; doi:10.1371/journal.pone.0067407)

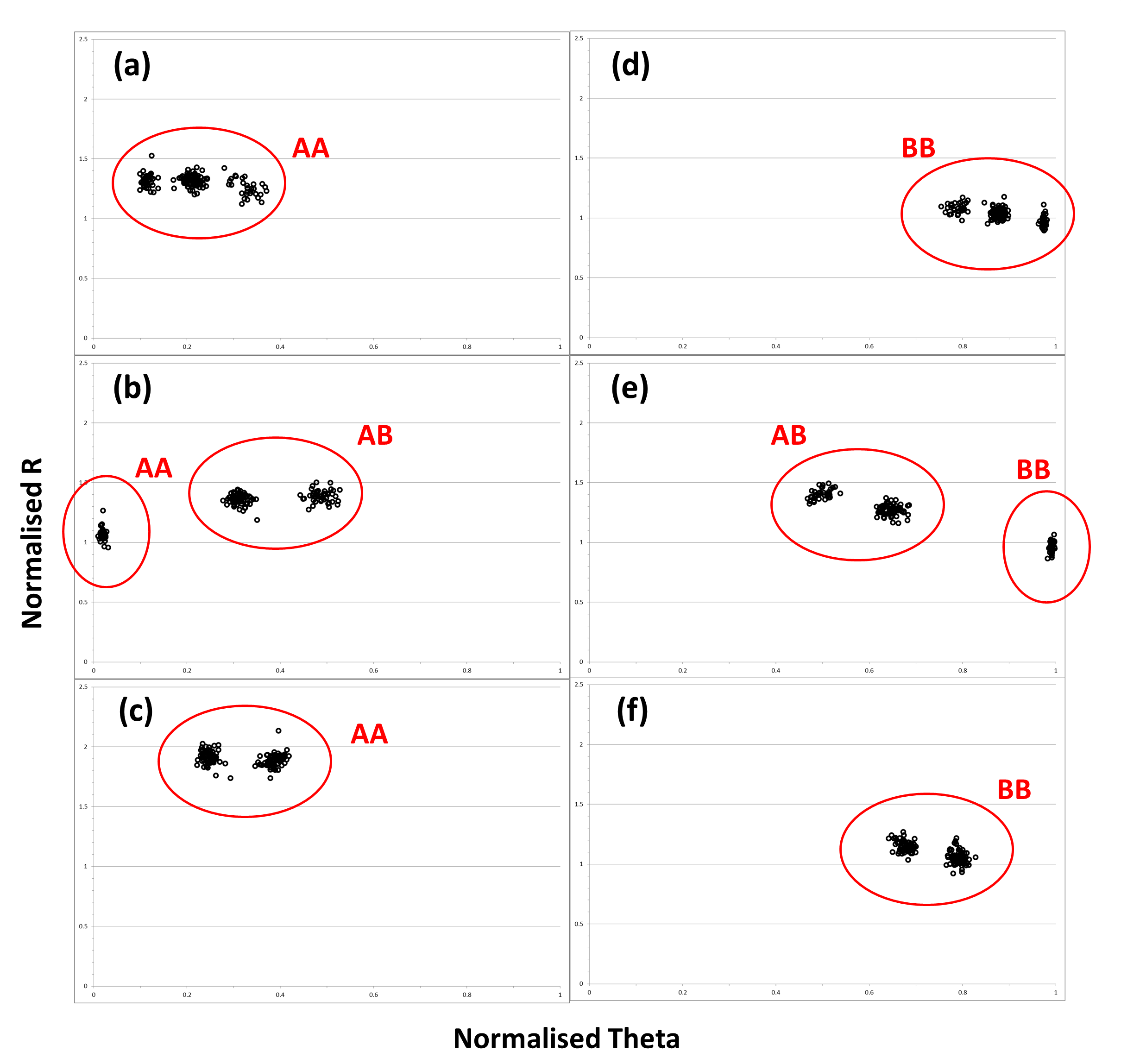

Supplement: Figure S1 — Erroneously scored genotype clusters annealing to multiple genomic regions. Examples of erroneously scored genotype clusters following automatic genotyping using GenomeStudio (Illumina)of SNP that target multiple sites. Red circles and genotypes written in red indicate genotype assignment by GenomeStudio: (a) Data scored as monomorphic AA clearly clustering in three genotype groups (AA/AA:AB/AA:BB/AA); (b) data scored as segregating 1∶1 (AA:AB) clearly segregating in three genotype groups (AA/AA:AB/AA:BB/AA); (c) Data scored as monomorphic AA clearly segregating in two genotype groups (AA/AA:AB/AA); (d) Data scored as monomorphic BB clearly clustering in three genotype groups (AA/BB:AB/BB:BB/BB); (e) data scored as segregating 1∶1 (AB:BB) clearly segregating in three genotype groups (AA/BB:AB/BB:BB/BB); (f) Data scored as monomorphic BB clearly segregating in two genotype groups (AB/BB:BB/BB). (PNG) [file pone.0067407.s001.png]

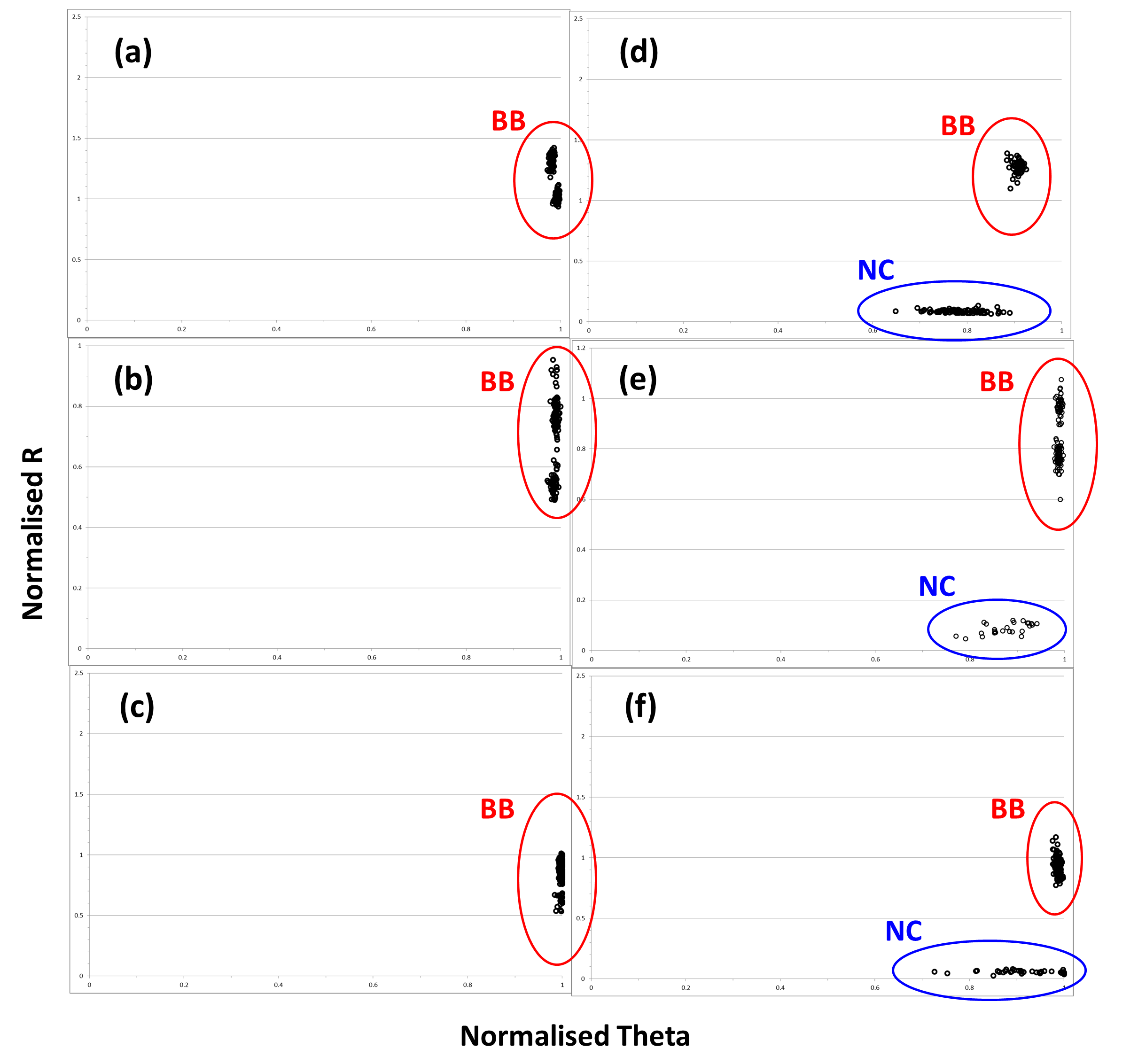

Supplement: Figure S2 — Erroneously scored genotype clusters containing additional SNPs. Examples of erroneously scored genotype clusters following automatic genotyping using GenomeStudio (Illumina) containing additional SNPs (hereafter reported as B’) and null (n) alleles. Red circles and genotypes indicate scores assigned by GenomeStudio, blue circles indicate genotypes assigned ‘no call’ (NC) by GenomeStudio: (a) Data scored as monomorphic BB clustering in two genotype groups (BB’:BB) resulting from BB’×BB; (b) Data scored as BB clustering in three genotype groups (BB:BB’:B’B’) resulting from BB’×BB’; (c) Data scored as BB clustering in two genotype groups (BB or BB’:B’B’) resulting from BB’×BB’; (d) Data scored as BB clustering in two genotype groups (Bn:nn) resulting from Bn×nn; (e) Data scored as BB clustering in three genotype groups (BB:Bn:nn) resulting from Bn×Bn; (f) Data scored as BB clustering in two genotype groups (BB or Bn:nn) resulting from Bn×Bn. (PNG) [file pone.0067407.s002.png]

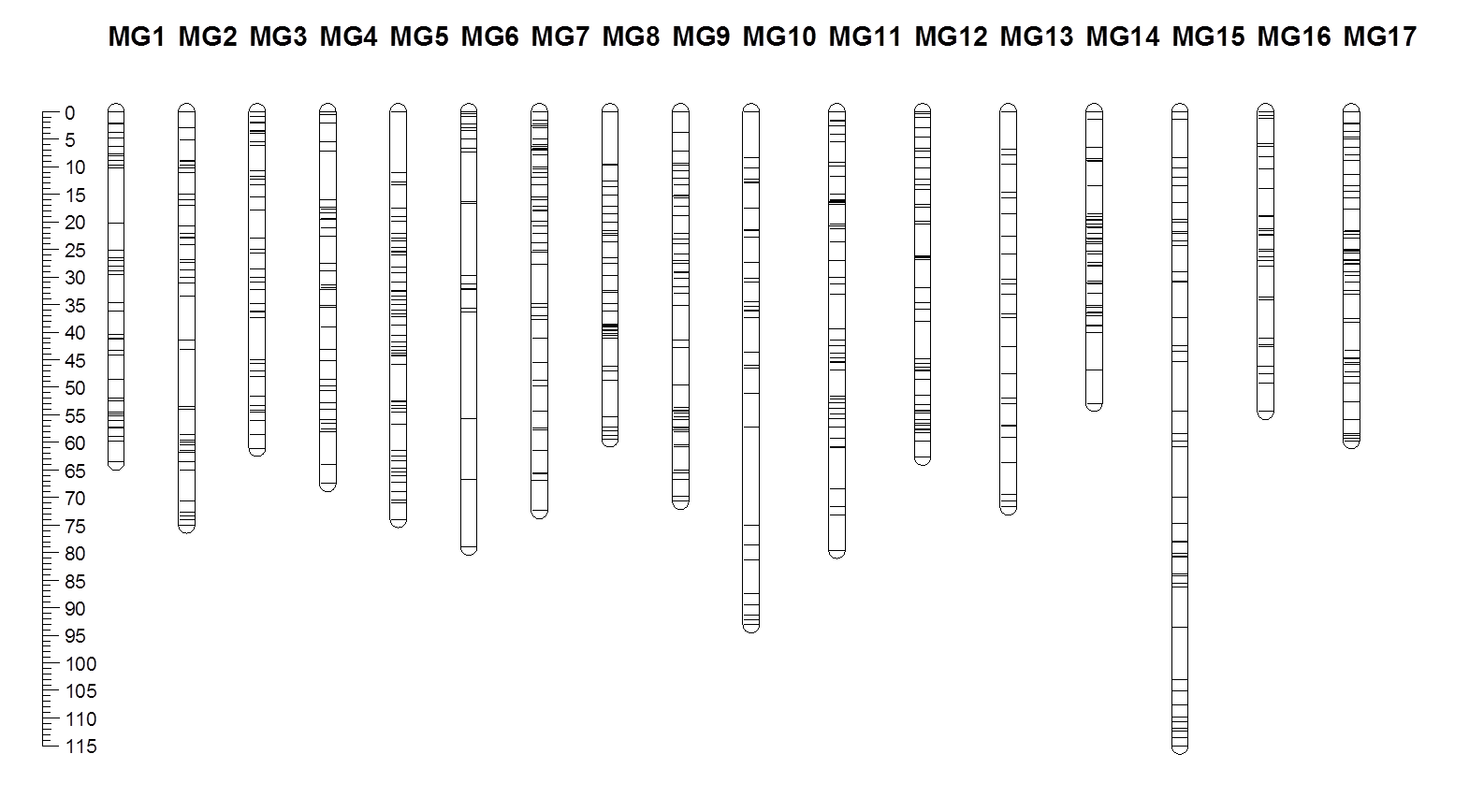

Supplement: Figure S3 — Linkage map of newly-mapped SNP markers. The M432 consensus linkage map detailing the map positions of the 797 novel SNP loci mapped in this investigation following manual re-annotation of data generated with the IRSC array. The scale in centi-Morgans (cM) is given at the left edge of the figure. (PNG) [file pone.0067407.s003.png]
