# Supplementary material for: Evaluation of SNP Data from the Malus Infinium Array Identifies Challenges for Genetic Analysis of Complex Genomes of Polyploid Origin
Source: PLoS One. 2013 Jun 27;8(6):e67407. doi: 10.1371/journal.pone.0067407 (PMC3694884; doi:10.1371/journal.pone.0067407)
Supplement: Table S1 — The names of the 192 Malus varieties genotyped with the IRSC array. (PDF) [file pone.0067407.s004.pdf]

Supplementary Table S1

Accession	Accession (continued)	Accession (continued)	Accession (continued)
Ace Spur	Cripps Red	Lalla Delicious	Red Haralson
Alka	Delblush	Lampone	Red Jacket
Alkmene	Delearly	Liberty	Red Miracle
Angold	Delicious	Limoncella	Redfree
Anna	Diwa	Limoncini	Regal Spur
Annurca	Djulabia	Macoun	Remo C
Aranciata di Cox	Dolgo	Magrè	Renoire
Arkansas	Domaine	Marcon	Resista
Arlet	Early Cortland	Mc Intosh	Richared Delicious
Babine	Early Red Stayman	Megumi	Rosemary Risset
Baldwin	Early Smith	Mela di Inverno	Rosmarina Rossa
Baujade	Eden Spur	Mela Forestiera	Royal Gala
Belchard Chantecler	Elite	Melrose	Royal Red Delicious
Big Red 1	Elstar	Monroe	Rubin
Boskoop	Emilia B9 1-28	Mosebar	Rubinette (Rafzubin)
Brina	Empire	Murray	Ruby
Calamari	Enterprise	Mutsu	Russian
Carola	Fiamma	Nabella	San Lugano
Cherrired	Florina	Nagano	Scotia
Cherry Cox	Fragoni	Newton Pippin	Selena
Clear Delicious	Fuji Mori Houfu 3a	Niagara	September
Clear Red	Fujion	Norten Spy	Seriana
Clivia	Gaia	Nova Easygro	Shelly
Close	Gallia Beauty	Okanagan	Shizuka
Commercio	Geneva	Okanoma	Ski Spur
Coop 1	Gloster	Oregon Spur	Smeralda
Coop 3	Goldchief	Oregon Spur	Smith Jonathan
Coop 6	Golden Orange	Orin	Spartan
Coop 7	Greendale	Orleans	Spokane Beauty
Coop 8	Haralson	Otterson	Stark Delicious
Coop 9	Heavy Stripe	Pacific Gold	Striato Dolce
Coop 10	Idagold	Painter Spur RD	Summerfree
Coop 11	Ingrid Maried	Paragon Winesap	Summerred
Coop 15 clone 1	Irish Peach	Parkman	Sunrise
Coop 15 clone 2	James Grieve	Peachblow	Superstarking D
Coop 16	Jerseymac	Petrel	Surprise
Coop 26	John Downie	PI 589746-05	Tavola Bianca
Coop 28	Jolly	Piattona	Top Crop
Coop 29	Jonafree	Pilot	Tsugaru
Coop 30	Jonagold	Pink Perl	Turner Delicious
Coop 36	Jonamac	Pirol-Pirella	Viking
Coop 38	Jonasty	Precoce Rigotti	Vistabella
Cornell Red	Jonathan	Priam	White Angel
Cortland	Jonica	Primiera	Wijcik Mcintosh
Cowichan	Kendal	Priscilla	William's Pride
Cox's Orange Pippin	Kidd's Orange Red	Ralls Janette	Winesap Spur
Crimson Beauty	Lady	Red Apple	Winter Winesap
Crimson Gold	Lady Williams	Red Flesh	Yellow Siberian
